# Vestibular Hypofunction in Patients with Presbycusis: A Cross-Sectional Study

**DOI:** 10.3390/jcm13247767

**Published:** 2024-12-19

**Authors:** Walid E. Omer, Khalid Abdulhadi, Saad Shahbal, Marcus Neudert, Timo Siepmann

**Affiliations:** 1 Audiology Unit, ENT Section, Ambulatory Care Center, Hamad Medical Corporation, Doha 3050, Qatar; 2 Dresden International University, 01067 Dresden, Germany; 3 Department of Medicine, Hamad Medical Corporation, Doha 3050, Qatar; 4 Department of Otorhinolaryngology, Head and Neck Surgery, Medical Faculty, University Hospital Carl Gustav Carus, TUD Dresden University of Technology, 01307 Dresden, Germany; 5 Department of Neurology, Medical Faculty, University Hospital Carl Gustav Carus, TUD Dresden University of Technology, 01307 Dresden, Germany

**Keywords:** presbycusis, vestibular hypofunction, hearing loss, vertigo, dizziness

## Abstract

**Background:** Vestibular Hypofunction (VH) and hearing loss can affect quality of life and lead to disability, especially in the elderly. Studies investigating presbycusis and vestibular function in the aging population have been conducted separately, but few have examined the combination of both conditions in older patients, with inconsistent results that may be due to small sample sizes or heterogeneity in the methods used to assess vestibular function. We aimed to characterize the occurrence of VH in patients with presbycusis using the video head impulse test (vHIT), which is a specific and reliable assessment tool for VH. **Methods:** A prospective, cross-sectional study of 200 individuals was conducted at Hamad Medical Corporation-Qatar in Doha, Qatar. Adults aged 50 years and older with bilateral age-related hearing loss were screened for eligibility and those who were eligible completed the vHIT. Patient history, demographics, audiological and clinical data were collected. VH was defined as vestibulo-ocular reflex (VOR) gain of <0.79 and presence of corrective saccades. **Results:** In our study population, (n = 200 mean age 64.1 ± 8.4 [Range = 37], 31.5% females), VH was identified in 12 out of 200 patients (6.0%, 95% CI: 3.3–10.1) with bilateral age-related hearing loss (ARHL), equivalent to approximately 1 in 17 patients. VH was detected in the left ear in 11 patients (5.5%, 95% CI: 2.9–9.9), the right ear in five patients (2.5%, 95% CI: 0.8–5.8), and bilaterally in four patients (2.0%, 95% CI: 0.6–5.4). **Conclusions:** In our study population, vestibular hypofunction was observed in 6% of patients with bilateral symmetrical age-related sensorineural hearing loss, suggesting that vestibular screening may be useful in this population at risk.

## 1. Introduction

Hearing is one of the five senses and is integral to human communication. The ear, as a hearing organ, is also responsible for balance through the vestibular system (sometimes referred to as the sixth sense), which functions to stabilize gaze, posture, and gait during movement [1]. Age-related hearing loss (ARHL) and vestibular hypofunction (VH) are two common sensory deficits in the elderly that can affect their quality of life and lead to disability [2,3,4,5]. Population-based studies have shown a decline in vestibular function with age [2,6]. It has also been reported that 43% of men and 20% of women aged 60–69 years in the United States have bilateral hearing loss [7], while in Europe at least 30% of the population aged over 55 years have ARHL [8]. The World Health Organization (WHO) estimates that by 2025, more than 500 million people will be affected by presbycusis [9]. As the world’s population continues to age, the economic costs associated with these conditions represent a significant burden on healthcare systems that will continue to increase without appropriate mitigation strategies [10,11]. This highlights the epidemiologic and economic burden of ARHL and VH in aging populations.

Presbycusis is defined as progressive bilateral symmetrical ARHL, usually confined to higher frequencies [12], and is the most common cause of sensory deficit in the elderly, and can be associated with several sociodemographic factors and health risks, including dementia, social isolation, depression, cardiovascular disease and falls [13,14]. A variety of mechanisms contribute to the development, onset and severity of presbycusis. In addition to aging, other risk factors include genetic susceptibility, noise exposure, ototoxic agents, metabolic disorders (e.g., obesity, hypertension, hyperglycaemia and dyslipidaemia), and lifestyle factors (e.g., smoking) [15]. VH in the elderly or age-related vestibular loss (ARVL), also known as presbyastasis or presbyequilibrium, impairs the vestibular system’s ability to maintain balance, often manifesting as dizziness, imbalance, oscillopsia, and an increased risk of falls [6], which are the leading cause of accidental death [16]. Both conditions contribute independently to morbidity in older adults, but their potential co-occurrence and association remains poorly understood.

There is a possible biological and genetic link between these two conditions. The cochlea and the saccule, key components of the auditory and vestibular systems, share a common embryonic origin—Otic Capsule [17]. This common developmental pathway raises the possibility that age-related degeneration affecting one system may also affect the other.

In addition, genetic studies have begun to shed light on the potential heritable components underlying these conditions. For example, research has begun to outline the role of common and rare variants in the human genome in vestibular disorders, suggesting overlapping mechanisms with hereditary forms of sensorineural hearing loss [18]. Advances in the genetics of vestibular disorders also point to individualized treatment approaches, particularly in conditions with expected comorbid auditory and vestibular dysfunction, such as Usher syndrome [19,20]. While clinical plausibility also supports this association, the potential relationship between these conditions remains underexplored, highlighting the gap in research into the prevalence and relationship between presbycusis and VH.

Previous studies investigating presbyacusis and vestibular function in the aging population have been conducted separately [21,22,23,24], few have investigated the combination of both conditions in the elderly with inconsistent results, possibly due to small sample sizes or the use of different methods of vestibular assessment with varying levels of specificity [25,26,27,28], with some of them basing the assessment tool on the occurrence of balance symptoms [29] or on a dizziness questionnaire [30]. A review by Parlu and colleagues [14] highlighted the need for research in this field and emphasized that epidemiological studies of the prevalence of vestibular hypofunction in the aging population, using standardized quantitative assessments of vestibular end-organ function, are high-impact research objectives that should be prioritized.

Filling this knowledge gap is important, because the coexistence of ARHL and VH could exacerbate functional impairment in the elderly, particularly in relation to fall risk and reduced mobility [2,3,4,5,6]. Understanding this association could guide more comprehensive diagnostic and therapeutic strategies, thereby improving care and quality of life for this vulnerable population.

The video head impulse test (vHIT) is a diagnostic tool for the accurate detection of vestibular hypofunction [31]. It has advantages over the standard head impulse test (HIT), such as differentiating peripheral from central vestibular loss—being positive in peripheral vestibulopathy. It is also readily available, less invasive and has a simpler setup compared to classical tests such as the scleral search coil [32]. In addition, vHIT is more sensitive and specific as it detects both overt and covert saccades, whereas bedside HIT only detects overt saccades [33]. The scleral search coil test (SSCT) is considered the gold standard test for vestibulo-oculomotor reflex (VOR) gain and corrective eye movement testing, and the vHIT is equivalent to the SSCT in identifying peripheral vestibular deficits [34].

We aimed to characterize the prevalence of VH in patients with presbycusis (ARHL) and to investigate their co-occurrence using the video head impulse test (vHIT), a specific and reliable tool for the assessment of vestibular function.

## 2. Materials and Methods

We performed an observational cross-sectional study conducted between January 2020 and June 2024 at the Audiology unit within the ENT Department of the Ambulatory Care Center (ACC) under Hamad Medical Corporation (HMC) in Qatar. The study was reviewed and approved by the Institutional Review Board of Hamad Medical Corporation and conducted in accordance with Good Clinical Practice guidelines and the guidelines of the Ministry of Public Health, Qatar.

### 2.1. Sample Size Determination

It was estimated that 35.4% of adults aged 40 years and older have vestibular dysfunction [2]. Based on clinical justification, a higher prevalence of vestibular hypofunction was anticipated (with a margin of error of ±7%).

To estimate the required sample size with a 95% confidence interval, the following formula was used [35]:

Sample size n = [DEFF * Np(1 − p)]/[(d^2^/Z^2_1 − α/2 * (N − 1) + p * (1 − p)]

where N is the population size, p is the estimated proportion, d is the precision of the estimate, and Z^2_1 − α/2 = 1.96.

Based on the equation, a sample size of 200 subjects was determined for this study.

### 2.2. Patient Selection

The study included adults aged 50 years and older with bilateral symmetric sensorineural hearing loss classified as mild to profound according to the American Speech-Language-Hearing Association (ASHA) criteria [36], which is consistent with the World Health Organization (WHO) Grade 1 to 4 classification of hearing impairment [37]. Inclusion criteria required patients to have a bilateral, symmetric, downward sloping sensorineural hearing loss with a threshold of 25 dB or greater, as determined by conventional audiometry at frequencies of 250, 500, 1000, 2000, 4000, 6000 and 8000 Hz [38]. To ensure the diagnosis of presbycusis and to exclude other causes, the onset of hearing loss had to occur after the age of 50 years to exclude congenital or early-onset hearing loss.

The diagnosis of presbycusis was based on three key factors: audiometric assessment, exclusion of other potential causes, and consideration of age. These diagnostic criteria were applied in the study’s inclusion and exclusion parameters to ensure the selection of participants with hearing loss consistent with the clinical and audiological characteristics of presbycusis.

Patients with hearing loss due to ototoxic exposure, conductive hearing loss, or childhood onset of hearing loss were excluded to be consistent with the diagnosis of presbycusis and to exclude other congenital or acquired causes of hearing loss. Patients with cervical spine disease, cervical spine fractures or acute spinal syndromes were also excluded as the video head impulse test (vHIT) is contraindicated in these cases.

Eligible patients were contacted by telephone and invited to visit the ENT department of the Ambulatory Care Center (ACC). Detailed informed consent forms were reviewed and provided to potential participants, outlining the patients’ rights and responsibilities throughout the research process. Participants who agreed to the research conditions and signed the informed consent underwent audiological testing (pure tone audiometry and tympanometry) to confirm eligibility.

### 2.3. Data Collection

A structured questionnaire was used to collect demographic and clinical data from the patients and their medical records were used to record risk factors.

### 2.4. Study Tests

Eligible participants underwent vHIT using ICS impulse video glasses (GN Otometrics^®^, ICS Charter vHIT device, OTOsuite vestibular software version: 4.10 Build 1341). The tests were performed by audiologists and/or audiology technicians who are licensed professionals and trained in various audiological tests, including hearing tests and vHIT audiologists and/or audiology technicians. The test consists of high-frequency head movements (“head impulses”) to both sides (right and left). During the test, the subject fixates a fixed point directly in front of them at a distance of at least 1 metre. Head impulses (more than 150 degrees per second) were applied randomly in the plane of the horizontal semicircular canals. Testing continued until 20 head impulses were completed for each side (right and left). Eye movements were recorded by the vHIT camera during the head impulses. Vestibular dysfunction results in corrective eye movements, called catch-up saccades, which are detected by the vHIT system in addition to VOR gain. Covert and overt saccades were analysed together. The primary outcome, VH, is defined as a VOR gain of less than 0.79 and the presence of corrective saccades [39]. Artefacts in saccades were reported as inconclusive. We used the three-frequency pure tone average (PTA) measured at 0.5, 1 and 2 kHz as the standard measure for calculating the pure tone average in our study [40,41].

### 2.5. Statistical Analysis

Descriptive statistics including counts (n), percentages (%) were calculated for the demographic and clinical characteristics of the patients. In addition, an independent samples *t*-test was used to compare whether there were differences in mean PTA and gain by laterality. In cases where assumptions failed, the non-parametric Mann–Whitney U test/Wilcoxon Signed Rank test was used. Chi-squared test was used to examine factors associated with PTA and VH.

## 3. Results

### 3.1. Study Participants

The study sample consisted of 200 patients classified as African, European/American, South/East Asian and West Asian according to the geographical location of the country. The majority of patients were male (n = 137, 68.5%) and aged 60–69 years (n = 83, 41.5%, 95% CI: 34.7–48.7). Despite being older, 104 of the patients were employed, the majority in white-collar jobs (n = 91, 45.5%, 95% CI: 38.5–42.7). The most common auditory symptom reported by subjects was tinnitus (n = 84, 42.0%, 95% CI: 35.1–49.2), followed by dizziness (n = 81, 40.5, 95% CI: 33.7–47.7). The most common comorbidities were diabetes mellitus (n = 94, 47.0%, 95% CI: 40.0–54.2), hypertension (n = 89, 44.5%, 95% CI: 37.5–51.7), and dyslipidaemia (n = 35, 17.5%, 95% CI: 12.6–23.6). More than 66% of patients (n = 133, 95% CI = 59.4–72.9) had two or more comorbidities (Table 1).

### 3.2. Auditory and Vestibular Disorders and Their Prevalence

VH was detected in 12 out of 200 patients (6.0%, 95% CI: 3.3–10.1) with bilateral age-related hearing loss (ARHL), equivalent to approximately 1 in 17 patients. VH was detected in the left ear in 11 patients (5.5%, 95% CI: 2.9–9.9), the right ear in five patients (2.5%, 95% CI: 0.8–5.8), and bilaterally in four patients (2.0%, 95% CI: 0.6–5.4).

The majority of patients had a PTA ≤ 55 dB in both the left ear (n = 186, 93.0%, 95% CI: 88.3–96.0) and the right ear (n = 182, 91.0%, 95% CI: 85.9–94.4). Saccades were observed in 15.5% of patients in both ears (n = 31, 95% CI: 10.9–21.4). Regarding their gain, most patients had a gain value of more than 0.79 in both the left ear (n = 179, 89.5%, 95% CI: 84.2–93.2) and the right ear (n = 193, 96.5%, 95% CI: 92.6–98.5). (Table 2).

A statistically significant difference was noted between the values of the right and left ears for both the PTA and the VOR gain test.

The mean PTA levels for the right and left ears were compared using the Wilcoxon Signed Rank Test, yielding statistically significant differences. However, these differences were not clinically significant. A similar pattern was observed for vHIT gain values, where the statistical significance did not translate into clinical relevance. (Table 3).

### 3.3. Factors Associated with Hearing Impairment and VH

PTA increased with patient age in both left and right ears. A statistically significant positive association was found between the number of comorbidities and PTA in the left ear, but no significant association was found in the right ear (Table 4). However, none of the risk factors tested had a statistically significant association with vestibular hypofunction (Table 5, Table 6 and Table 7).

To note, PTA values below 25 dB were still consistent with the diagnosis of presbycusis, as hearing loss in these individuals was limited to higher frequencies (4–8 kHz). Additionally, the PTA (in dB HL) data were divided into five categories rather than being dichotomized into >55 dB or <55 dB, allowing for more detailed analysis.

## 4. Discussion

The main finding of our study is that VH was observed in 1 out of 17 patients with bilateral ARHL, suggesting that vestibular screening may be helpful in this at-risk population. VH was more frequently detected in the left ear (5.5%) compared to the right ear (2.5%), with (2.0%) of patients with bilateral VH.

Aging is a natural process that gradually diminishes several organ systems, including the sensory end organs of the inner ear—both vestibular and auditory—where both structures show age-related changes in structure and function. Similar age-related processes that can cause auditory end-organ loss can also affect the vestibular end-organ, resulting in age-related vestibular loss (ARVL) or hypofunction. However, the mechanisms linking age-related hearing loss (ARHL) and vestibular hypofunction remain complex and multifactorial, with individual differences in the expression and effects of these factors. These factors may be intrinsic, such as genetic predisposition, in addition to extrinsic influences which include medications (ototoxic agents), environmental factors (noise exposure), comorbidities (such as cardiovascular disease) and lifestyle influences (smoking). The underlying pathology of these age-related auditory and vestibular changes involves various processes such as inflammation, age-related oxidative stress and genetic contributions [14].

McGarvie and colleagues 2015 [21] found no decline in VOR function with age, as measured by vHIT in healthy subjects across different age groups. Similarly, a review of studies on caloric testing across different healthy age groups found no deterioration of VOR function with age [22]. In contrast, our results found a 6% (1 in 17) prevalence of VH in patients with ARHL. Notably, our study included only patients with ARHL, unlike the previously mentioned studies [21,22] which focused on healthy subjects. Say and colleagues 2021 found that right and left lateral VOR gain scores were reduced in the presbycusis group compared to the control group, which is similar to our study findings [26]. However, the smaller sample size of 20 subjects in their study (in the study arm) may limit generalisability, nevertheless their findings are consistent with the patterns observed in our cohort. Shubert and colleagues [27] examined 185 patients with CPA tumours using the caloric test and video head impulse test, the majority of whom were over 50 years of age (mean age 62.3 ± 12.6 years), and found that high frequency hearing loss did not predict VOR hypofunction; however, an important source of variability in the results may be the inclusion of patients with high frequency hearing loss secondary to CPA tumours in their study, whereas our study focused on age-related hearing loss.

In contrast, the prevalence of VH and presbycusis in a study population of 216 participants was 30.1%, but they used the modified clinical test of sensory interaction on balance [25] as an assessment tool to diagnose vestibular dysfunction. Another study was conducted in Malaysia and found the prevalence of vestibular dysfunction to be 27% [28]. However, this study was limited by a small sample size. Moreover, the cut-off for diagnosing vestibular dysfunction was not defined in this study. In addition, a subjective assessment tool—the Malay Version Vertigo Symptoms Scale—was also used, which may increase the percentage of vestibular dysfunction, although it was not clear which side was affected. Our use of the vHIT provided an objective measure of vestibular function [42]. The differences in the results among studies may be attributed to differences in study populations and methods. In addition, the definition of VH in different studies was another source of variability in estimating the prevalence of VH.

While our study highlights the prevalence of VH in patients with ARHL, the lack of a control group limits our ability to establish a direct association between these conditions. Further controlled studies are needed to better understand the direct relationship between ARHL and VH. Another limitation of our study is the lack of a full vestibular test battery, such as videonystagmography, caloric testing, and kinetic rotatory chair testing, to further confirm the absence of vestibular dysfunction.

No specific associated factor was identified to increase the risk of VH, probably due to its relatively low detected prevalence. Therefore, regression modelling was not performed in our study as it is unlikely to identify a meaningful association. Another notable observation from our study is the asymmetric distribution of VH, with the left ear being more commonly affected compared to the right, which is consistent with the findings of Schubert et al. [27]. The reasons for this asymmetry remain unclear and may represent a possible point of investigation in the future. The utility of vHIT in the assessment of acute and chronic vertigo disorders warrants further investigation, especially in the at-risk population of elder patients with hearing impairment and those with common neurological disorders like cerebrovascular disease [34,43,44].

## 5. Conclusions

In our cohort analysis, vestibular hypofunction was observed in 6% of patients with bilateral age-related symmetric sensorineural hearing loss, suggesting that vestibular screening may be useful in this at-risk population. Future research should focus on controlled and longitudinal studies to further clarify the relationship between ARHL and VH and to develop targeted interventions.

## Figures and Tables

**Table 1:** Demographic profile of the patients (N = 200).

Category	Factors	N (%)
Gender	Male	137 (68.5)
	Female	63 (31.5)
Age	50–64	110 (55)
	>64	90 (45)
Nationality	African	47 (23.5)
	Europe/American	9 (4.5)
	South/East Asian	19 (9.5)
	West Asian	125 (62.5)
Occupation	Blue color job	13 (6.5)
	White color job	91 (45.5)
	Retired	96 (48)
Chronic diseases	No chronic disease	18 (9)
	1 chronic disease	49 (14.5)
	≥2 chronic disease	133 (66.5)
	Hypertension	89 (44.5)
	Diabetes mellitus	94 (47)
	Dyslipidemia	35 (17.5)
	Cardiovascular disease	16 (8)
	Thyroid dysfunction	15 (7.5)
	Others *	27 (13.5)
Associated symptoms	Dizziness	81 (40.5)
	Tinnitus	84 (42)

* Others chronic diseases include Anemia, Cancer, Prostatitis, Chronic kidney disease, Thalassemia.

**Table 2:** Prevalence of the hearing impairments and VH.

Variable	Category	N (%)
PTA_left	≤55	186 (93)
	>55	14 (7)
PTA_right	≤55	182 (91)
	>55	18 (9)
Saccade left head impulses	Inconclusive	4 (2)
	Normal/Absent	165 (82.5)
	Saccade/present	31 (15.5)
Saccade right head impulses	Inconclusive	5 (2.5)
	Normal/Absent	164 (82)
	Saccade/present	31 (15.5)
Gain_left	<0.79	21 (10.5)
	≥0.79	179 (89.5)
Gain_right	<0.79	7 (3.5)
	≥0.79	193 (96.5)
VH_left	Inconclusive *	33 (16.5)
	Normal	156 (78)
	VH	11 (5.5)
VH_right	Inconclusive *	33 (16.5)
	Normal	162 (81)
	VH	5 (2.5)

* Inconclusive VH: either having decreased gain only or saccades only, but not both together to qualify for VH.

**Table 3:** PTA (in dB HL) and vHIT gain based on laterality.

Variables	Laterality	Mean ± SD	Shapiro-Wilk Normality *p*-Value	Wilcoxon Signed Rank Test *p*-Value
PTA	Right (Range: 85)	33.8 ± 16.4	<0.01	<0.01
	Left (Range: 72)	32.4 ± 15.5	<0.01	
Gain	Right (IQR *: 0.98 (0.91–1.07)	0.99 ± 0.2	<0.01	<0.01
	Left (IQR *: 0.91 (0.85–1.01)	0.93± 0.1	<0.01	

* IQR: Interquartile range.

**Table 4:** Factors associated with PTA.

Factor	Category	PTA_L					PTA_R				
		≤20 (n = 47)	21–40 (n = 90)	41–55 (n = 49)	56–70 (n = 12)	71–90 (n = 2)	≤20 (n = 51)	21–40 (n = 80)	41–55 (n = 51)	56–70 (n = 14)	71–90 (n = 4)
Gender	Male	9 (19.1)	33 (36.7)	16 (32.7)	4 (33.3)	1 (50.0)	11 (21.6)	30 (37.5)	13 (25.5)	7 (50.0)	2 (50.0)
	Female	38 (80.9)	57 (63.3)	33 (67.3)	8 (66.7)	1 (50.0)	40 (78.4)	50 (62.5)	38 (74.5)	7 (50.0)	2 (50.0)
	*p* value	0.31					0.12				
Age	50–64	9 (19.1)	33 (36.7)	16 (32.7)	4 (33.3)	1 (50.0)	11 (21.6)	30 (37.5)	13 (25.5)	7 (50.0)	2 (50.0)
	>64	38 (80.9)	57 (63.3)	33 (67.3)	8 (66.7)	1 (50.0)	40 (78.4)	50 (62.5)	38 (74.5)	7 (50.0)	2 (50.0)
	*p* value	<0.01					<0.01				
Occupation	Blue	3 (6.4)	5 (5.6)	3 (6.1)	2 (16.7)	0 (0.0)	3 (5.9)	4 (5.0)	4 (7.8)	2 (14.3)	0 (0.0)
	White	35 (74.5)	37 (41.1)	16 (32.7)	2 (16.7)	1 (50.0)	39 (76.5)	33 (41.3)	15 (29.4)	2 (14.3)	2 (50.0)
	Retired/Stay at home	9 (19.1)	48 (53.3)	30 (61.2)	8 (66.7)	1 (50.0)	9 (17.6)	43 (53.8)	32 (62.7)	10 (71.4)	2 (50.0)
	*p* value	0.31					0.12				
Nationality	African	13 (27.7)	16 (17.8)	16 (32.7)	1 (8.3)	1 (50.0)	15 (29.4)	14 (17.5)	14 (27.5)	3 (21.4)	1 (25.0)
	European/American	4 (8.5)	3 (3.3)	2 (4.1)	0 (0.0)	0 (0.0)	3 (5.9)	4 (5.0)	2 (3.9)	0 (0.0)	0 (0.0)
	South/East Asian	7 (14.9)	10 (11.1)	2 (4.1)	0 (0.0)	0 (0.0)	8 (15.7)	8 (10.0)	3 (5.9)	0 (0.0)	0 (0.0)
	West Asian	23 (48.9)	61 (67.8)	29 (59.2)	11 (91.7)	0 (0.0)	25 (49.0)	54 (67.5)	32 (62.7)	11 (78.6)	3 (75.0)
	*p* value	0.20					0.58				
Chronic disease	0	4 (8.5)	5 (5.6)	7 (14.3)	0 (0.0)	2 (100.0)	3 (5.9)	6 (7.5)	6 (11.8)	1 (7.1)	1 (33.3)
	1	12 (25.5)	22 (24.4)	14 (28.6)	1 (8.3)	0 (0.0)	13 (25.5)	21 (26.3)	13 (25.5)	2 (14.3)	0 (0.0)
	≥2	31 (66.0)	63 (70.0)	28 (57.1)	11 (91.7)	0 (0.0)	35 (68.6)	53 (66.3)	32 (62.7)	11 (78.6)	2 (66.7)
	*p* value	<0.01					0.12				

**Table 5:** Factors associated with vHIT gain.

Factor	Category	Gain_L		Gain_R	
		<0.79 (n = 21)	≥0.79 (n = 179)	<0.79 (n = 7)	≥0.79 (n = 193)
Gender	Male	10 (47.6)	100 (55.9)	3 (42.9)	107 (55.4)
	Female	11 (52.4)	79 (44.1)	4 (57.1)	86 (44.6)
	*p* value	0.29		0.16	
Age	50–64	10 (47.6)	100 (55.9)	3 (42.9)	107 (55.4)
	>64	11 (52.4)	79 (44.1)	4 (57.1)	86 (44.6)
	*p* value	0.63		0.79	
Occupation	Blue	2 (9.5)	11 (6.1)	1 (14.3)	12 (6.2)
	White	9 (42.9)	82 (45.8)	3 (42.9)	88 (45.6)
	Retired/Stay at home	10 (47.6)	86 (48.0)	3 (42.9)	93 (48.2)
	*p* value	0.83		0.69	
Nationality	African	4 (19.0)	43 (24.0)	1 (14.3)	46 (23.8)
	European/American	0 (0.0)	9 (5.0)	0 (0.0)	9 (4.7)
	South/East Asian	0 (0.0)	19 (10.6)	1 (14.3)	18 (9.3)
	West Asian	17 (81.0)	108 (60.3)	5 (71.4)	120 (62.2)
	*p* value	0.19		0.84	
Chronic disease	0	0 (0.0)	18 (10.1)	0 (0.0)	18 (9.3)
	1	6 (28.6)	43 (24.0)	3 (42.9)	46 (23.8)
	≥2	15 (71.4)	118 (65.9)	4 (57.1)	129 (66.8)
	*p* value	0.31		0.42	

**Table 6:** Factors associated with Saccades.

		Saccade Left Head Movement			Saccade Right Head Movement		
Factor	Category	Inconclusive (n = 4)	Normal (n = 165)	Saccade (n = 31)	Inconclusive (n = 5)	Normal (n = 164)	Saccade (n = 31)
Gender	Male	2 (50.0)	55 (33.3)	6 (19.4)	2 (40.0)	55 (33.5)	6 (19.4)
	Female	2 (50.0)	110 (66.7)	25 (80.6)	3 (60.0)	109 (66.5)	25 (80.6)
	*p* value	0.22			0.27		
Age	50–64	2 (50.0)	95 (57.6)	13 (41.9)	2 (40.0)	95 (57.9)	13 (41.9)
	>64	2 (50.0)	70 (42.4)	18 (58.1)	3 (60.0)	69 (42.1)	18 (58.1)
	*p* value	0.27			0.21		
Occupation	Blue	1 (25.0)	10 (6.1)	2 (6.5)	1 (20.0)	8 (4.9)	4 (12.9)
	White	1 (25.0)	74 (44.8)	16 (51.6)	0 (0.0)	77 (47.0)	14 (45.2)
	Retired/Stay at home	2 (50.0)	81 (49.1)	13 (41.9)	4 (80.0)	79 (48.2)	13 (41.9)
	*p* value	0.55			0.10		
Nationality	African	3 (75.0)	37 (22.4)	7 (22.6)	2 (40.0)	39 (23.8)	6 (19.4)
	European/American	0 (0.0)	8 (4.8)	1 (3.2)	0 (0.0)	8 (4.9)	1 (3.2)
	South/East Asian	0 (0.0)	19 (11.5)	0 (0.0)	0 (0.0)	16 (9.8)	3 (9.7)
	West Asian	1 (25.0)	101 (61.2)	23 (74.2)	3 (60.0)	101 (61.6)	1 (3.2)
	*p* value	0.10			0.93		
Chronic disease	0	0 (0.0)	16 (9.7)	2 (6.5)	0 (0.0)	18 (11.0)	0 (0.0)
	1	0 (0.0)	41 (24.8)	8 (25.8)	2 (40.0)	39 (23.8)	8 (25.8)
	≥2	4 (100.0)	108 (65.5)	21 (67.7)	3 (60.0)	107 (65.2)	23 (74.2)
	*p* value	0.66			0.30		

**Table 7:** Factors associated with VH.

		Left			Right		
Factor	Category	N/I (n = 33)	Normal (n = 156)	VH (n = 11)	N/I (n = 33)	Normal (n = 162)	VH (n = 5)
Gender	Male	8 (24.2)	53 (34.0)	2 (18.2)	8 (24.2)	55 (34.0)	0 (0.0)
	Female	25 (75.8)	103 (66.0)	9 (81.8)	25 (75.8)	107 (66.0)	5 (100.0)
	*p* value	0.34			0.17		
Age	50–64	14 (42.4)	91 (58.3)	5 (45.5)	12 (36.4)	95 (58.6)	3 (60.0)
	>64	19 (57.6)	65 (41.7)	6 (54.5)	21 (63.6	67 (41.4)	2 (40.0)
	*p* value	0.20			0.06		
Occupation	Blue	3 (9.1)	9 (5.8)	1 (9.1)	4 (12.1)	8 (4.9)	1 (20.0)
	White	13 (39.4)	72 (46.2)	6 (54.5)	11 (33.3)	77 (47.5)	3 (60.0)
	Retired/Stay at home	17 (51.5)	75 (48.1)	4 (36.4)	18 (54.5)	77 (47.5)	1 (20.0)
	*p* value	0.83			0.19		
Nationality	African	9 (27.3)	36 (23.1)	2 (18.2)	7 (21.2)	39 (24.1)	1 (20.0)
	European/American	1 (3.0)	8 (5.1)	0 (0.0)	1 (3.0)	8 (4.9)	0 (0.0)
	South/East Asian	0 (0.0)	19 (12.2)	0 (0.0)	2 (6.1)	16 (9.9)	1 (20.0)
	West Asian	23 (69.7)	93 (59.6)	9 (81.8)	23 (69.7)	99 (61.1)	3 (60.0)
	*p* value	0.27			0.93		
Chronic disease	0	2 (6.1)	16 (10.3)	0 (0.0)	0 (0.0)	18 (11.1)	0 (0.0)
	1	10 (30.3)	37 (23.7)	2 (18.2)	11 (33.3)	37 (22.8)	1 (20.0)
	≥2	21 (66.0)	103 (66.0)	9 (81.8)	22 (66.7)	107 (66.0)	4 (80.0)
	*p* value	0.61			0.23		

N/I: Inconclusive: either having decreased gain only or saccades only but not both together to qualify for VH.

## Data Availability

The original contributions presented in this study are included in the article. Further inquiries can be directed to the corresponding author.
